# The West Dallas environmental health project: the importance of community health experiences related to air pollution

**DOI:** 10.3389/fpubh.2025.1613899

**Published:** 2025-07-25

**Authors:** Xiara Favorite, Janie Cisneros, Alicia Kendrick, Misti O’Quinn, Evelyn Mayo, Caleb Roberts, Natalie Johnson, Ping Ma

**Affiliations:** 1 Department of Environmental and Occupational Health, Texas A&M University School of Public Health, College Station, TX, United States; 2 Singleton United/Unidos, Dallas, TX, United States; 3 Downwinders at Risk, Good-Latimer, Dallas, TX, United States; 4 Department of Health Behavior, Texas A&M University School of Public Health, College Station, TX, United States

**Keywords:** fine particulate matter, community-based participatory research, community health survey, low-cost sensors, environmental justice

## Abstract

Racial and ethnic minorities experience a disproportionate exposure to air pollutants, such as fine particulate matter (PM_2.5_) and nitrogen dioxide (NO_2_), due to historical racial zoning increasing in proximity to industrial facilities. PM_2.5_ is associated with several adverse health effects including cardiopulmonary diseases, lung cancer, and adverse birth outcomes. Hence, reducing pollution exposure in minority communities, i.e., environmental justice (EJ) communities, holds great promise for reducing disparities in associated health burdens and improving health equity. In Dallas, Texas, residents living in an EJ community known as the “Singleton Corridor,” formed an action group to spread awareness of local pollution risks. Academic and community partners initiated a pilot study using a community-based participatory research (CBPR) approach, wherein volunteers administered a household survey from July to December 2023. Additionally, neighborhood-level PM_2.5_ concentration data from low-cost sensors were publicly accessible from the University of Texas at Dallas’ SharedAirDFW network and the City of Dallas. A total of 86 households completed the community survey. The majority of survey respondents (60.5%) rated the air quality as low or very low. 83.7% of respondents reported that air pollution in their neighborhood had made them or their family members sick. More than 60% of participants reported they avoid exercising outdoors and opening their windows due to concern about air pollution. 31.4% of respondents reported a lifetime diagnosis of asthma, with 26.7% reporting current asthma. Air monitoring data indicated potential PM_2.5_ hotspots necessitating further inquiry. Overall, the findings from this study indicate significant community concerns about air pollution exposure and a high prevalence of asthma.

## 1 Introduction

Despite substantial improvements in air quality in the U. S. since implementation of the Clean Air Act, millions of Americans, ~140 million people in 2023, live in counties with pollution levels above the National Ambient Air Quality Standards (1). It is well-established that racial and ethnic minorities experience a disproportionate exposure to air pollutants, such as fine particulate matter (PM_2.5_) and nitrogen dioxide (NO_2_), due to historical local- to national-scale mechanisms, including racist laws and actions (2, 45, 47). Kerr et al. (49) reported racial and ethnic disparities in NO_2_-attributable pediatric asthma and PM_2.5_-attributable premature mortality have widened in the U. S. during the last decade. Hence, reducing pollution exposure in marginalized communities, i.e., environmental justice (EJ) communities, holds great promise for reducing disparities in associated health burdens and improving health equity.

## 2 Context

**Figure 1:**
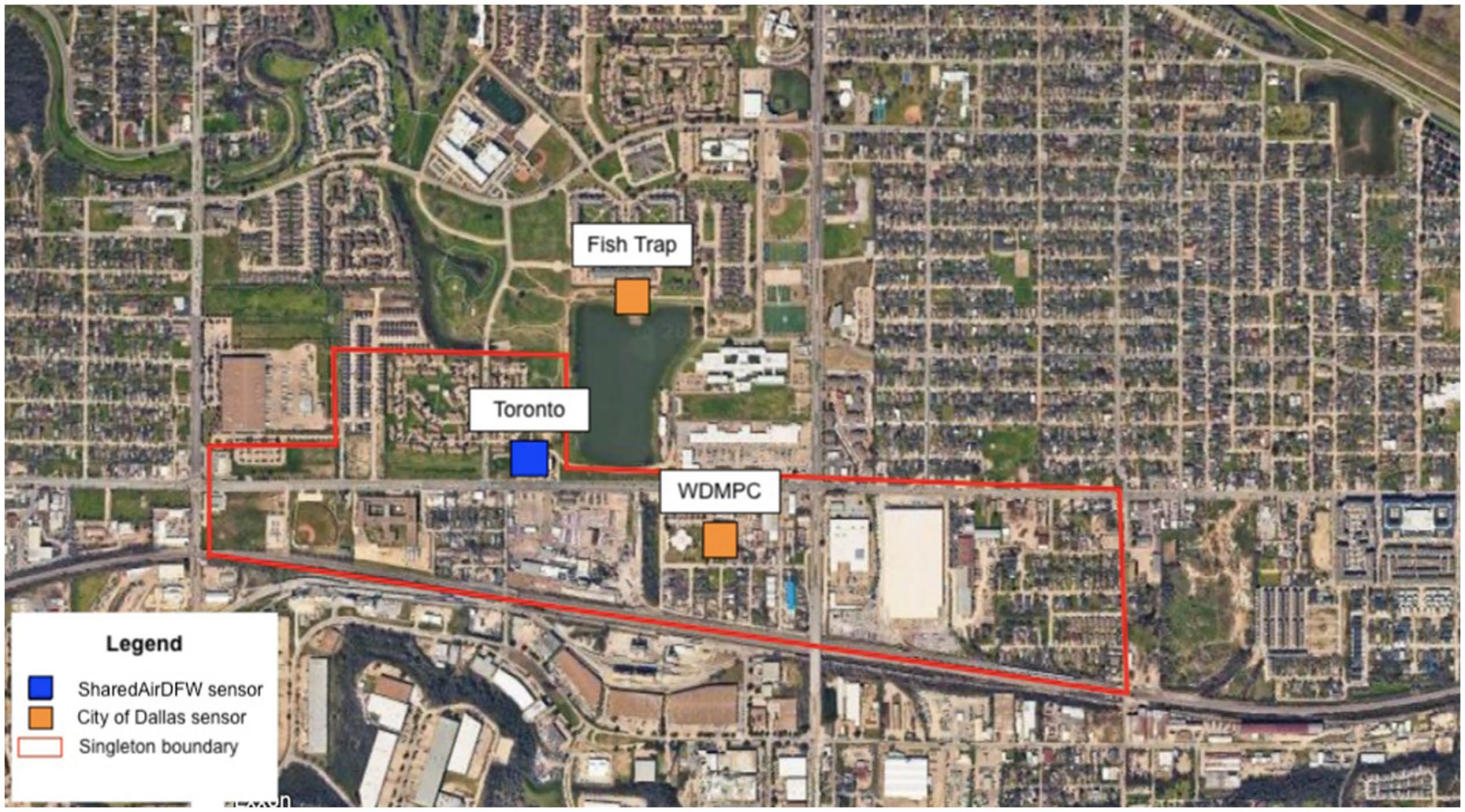
Map of the Singleton Corridor, West Dallas, TX. The blue squares and orange squares represent low-cost PM_2.5_ sensors from the SharedAirDFW Network and the City of Dallas, respectively.

West Dallas has a longstanding history of racial and environmental injustices that have shaped the community’s current conditions. In 1937, West Dallas was labeled a fourth-grade, “hazardous” area on the City of Dallas’ Residential Security Map—part of the federally sponsored redlining maps created by the Home Owner’s Loan Corporation (3). This designation was based not on housing quality or infrastructure but on the racial and ethnic composition of the neighborhood, which was predominantly Black and immigrant families. Despite noting that the area supported civic institutions such as schools and churches, this classification denied residents access to home loans and encouraged the expansion of local industrial uses—including concrete and asphalt plants—that eventually dominated the neighborhood. These discriminatory policies formalized environmental and economic disinvestment, contributing to the cumulative burdens residents face today. In the 1980s, elevated blood lead levels were measured in children, and many West Dallas residents reported health concerns in connection with the RSR Smelter (50), which was subsequently placed on the EPA’s Superfund List (48). Additionally, between 1967 and 1992, more than 396,000 tons of vermiculite containing asbestos was transported to the West Dallas W. R. Grace factory (4). Today, residents remain surrounded by sources of air pollution due to legacy industrial zoning. An EJ community within West Dallas known as the “Singleton Corridor,” consisting of residents who live south of Singleton Blvd. and in Kingbridge Crossing (corresponding to Census Tract 205: GEOID: 48113020500), formed an action group known as Singleton United/Unidos to spread awareness of local pollution risks (Figure 1). According to the U. S. Climate Vulnerability Index (5), this census tract ranks in the 96th percentile nationally for cumulative environmental, social, economic, and infrastructure vulnerabilities. Moreover, it is ranked in the 99th percentile for “community baseline” vulnerability, which captures long-standing inequities that shape resilience to climate impacts. Notably, Census Tract 205 is ranked 15th out of 5,265 census tracts in Texas, indicating one of the highest vulnerability levels statewide. In past community meetings, residents reported black soot covering their cars and expressed concern about chemical exposure from nearby industry. A map of Toxic Releases Inventory (TRI) facilities in the Singleton Corridor can be found in Supplementary Figure 1. By applying a community-based participatory research (CBPR) approach previously employed in a south Dallas EJ community known as Joppa (6), a steering committee led engagement events and organized volunteers to administer a household survey from July to December 2023. Additionally, neighborhood-level PM_2.5_ concentration data from low-cost sensors were publicly accessible from the University of Texas at Dallas SharedAirDFW network and the City of Dallas (Supplementary Figure 2). The main objective of this pilot study was to determine community perceptions of air quality and assess general health status through an established household survey.

## 3 Methods and results

Members from Singleton United/Unidos worked with academic researchers and a Dallas EJ grassroots organization, Downwinders at Risk, to adapt a community health survey (6) and recruit resident volunteers to administer the survey using a door-to-door canvassing method. Volunteers received training through the Collaborative Institutional Training Initiative (CITI) and all protocols and materials were approved through the Texas A&M University Institutional Review Board. The survey was offered online or via paper in both English and Spanish. Survey participants received a $10 gift card following completion of the survey. A total of 86 households participated in the survey. The majority of respondents were female (71.8%), racial/ethnic minorities (Black 84.7% and Hispanic 15.3%), aged 50 or older (63.5%), single without living with a partner (55.3%), non-smokers (55.8%), and had a high school or equivalent education (44.7%). Additional sociodemographic characteristics are detailed in Supplementary Table S1.

Supplementary Table S2 shows respondents’ perceived air pollution exposure and related concerns. Concerns about air quality in the Singleton Corridor were widespread, with 60.5% of participants rating it as low or very low, and an additional 27% considering it fair. Furthermore, 40.7% of respondents reported that the air quality inside their homes was poor or fair. A significant portion of participants indicated behavioral changes due to air pollution, with 61.6% avoiding outdoor exercise and 68.6% refraining from opening windows. A substantial number (88.4%) agreed or strongly agreed that air pollution in the neighborhood is indeed a problem, and 84.9% of participants believed nearby industries contributed to this issue. 83.7% of participants strongly agreed or agreed that air pollution in the neighborhood affected their health or their family’s health.

Participants reported high levels of exposure to different pollution sources, including traffic (59.3%), factories/smokestacks (79.1%), and trains (66.3%). Furthermore, 62.8% of respondents were moderately or extremely concerned that air pollution from factories will cause health problems. A substantial number (87.2%) believed factory pollution played a role in their health issues, followed by 62.63% attributing it to traffic, 68.6% to trains, and 5.8% to other sources. As high as 62.8% of respondents believed that air pollution may have made asthma and other respiratory diseases worse, and half of respondents believed it aggravated allergic reactions. 72.1% of respondents reported they believed air pollution increased difficulty breathing, 65.1% for Cough/Cold, and 62.8% for asthma and other respiratory disease.

Health status information (Supplementary Table S3) was based on a list of validated questions from previous studies (7, 8). A total of 31.4% of respondents reported having a lifetime diagnosis of asthma, with 26.7% of current asthma rate. Approximately 44.2% of respondents were identified as having a medium or high risk of developing Chronic Obstructive Pulmonary Disease (COPD), and 41.9% reported experiencing symptoms of respiratory diseases within the past 12 months. Additionally, 76.7% of respondents perceived moderate to high levels of stress. The survey also indicated that certain demographic subgroups were at higher risk for respiratory diseases. Specifically, female respondents were more likely to have an asthma diagnosis than males. Individuals in older age groups, those with lower educational attainment, those who were obese, single, and Black/African American respondents exhibited a higher risk of COPD.

From July to December 2023, real-time PM_2.5_ data were publicly accessible from various low-cost sensors, including the SharedAirDFW Network—a regional, hyper-local air monitoring system developed and maintained by the University of Texas at Dallas (UTD). These monitors utilize IPS Series Sensors (model IPS7100) manufactured by Piera Systems Inc. (2022), which are equipped with optical particle counting (OPC) technology and adjustable sensitivity controls to improve precision. The IPS7100 provides real-time PM_2.5_ concentration and particle count data every 30 s and is factory-calibrated with ±10% accuracy against Federal Equivalent Method (FEM) monitors. Data from SharedAirDFW sensors are displayed on a publicly accessible, interactive dashboard.

Sampling data collected from the SharedAirDFW Toronto St. monitor in West Dallas were cleaned and processed using RStudio Version 2024.04.2. Data points with missing PM_2.5_, temperature, or relative humidity (RH) values were removed. A correction factor was applied using a multiple linear regression (MLR) model developed by Raheja et al. (9), which adjusts for raw PM_2.5_ concentrations, RH, and temperature. This model was chosen based on its suitability for OPC-based monitors using BME280 sensors and its demonstrated ability to reduce RH-induced overestimation and sensor noise under varied environmental conditions.

Exceptionally high PM_2.5_ values were included in the cleaned dataset as long as their associated temperature and humidity values fell within plausible ranges. Specifically, we removed records with extreme temperature readings (< −100°F or > 500°F) and invalid relative humidity values (< 0% or > 100%) prior to correction and averaging. However, we did not apply a separate exclusion criterion based solely on PM2.5 concentration values, as these may reflect real short-term pollution events in highly industrial areas like West Dallas.

After quality control filtering, PM_2.5_ data were corrected using a multiple linear regression model developed by Raheja et al. (9), which accounts for temperature and humidity effects on sensor performance. The final corrected values were then aggregated to compute daily averages. As such, outliers were not excluded based solely on magnitude but were averaged in the daily means if they passed environmental plausibility checks and were not associated with sensor or data anomalies.

Although no formal co-location study was conducted in West Dallas, prior deployments of co-located PurpleAir and SharedAirDFW sensors in South Dallas (Joppa) demonstrated moderate to strong correlation between corrected sensor data (*r* = 0.66) and strong agreement with a nearby regulatory-grade monitor (*r* = 0.80–0.81). These findings, from a pending study, offer methodological support for interpreting the West Dallas sensor data using similar correction strategies.

**Figure 2:**
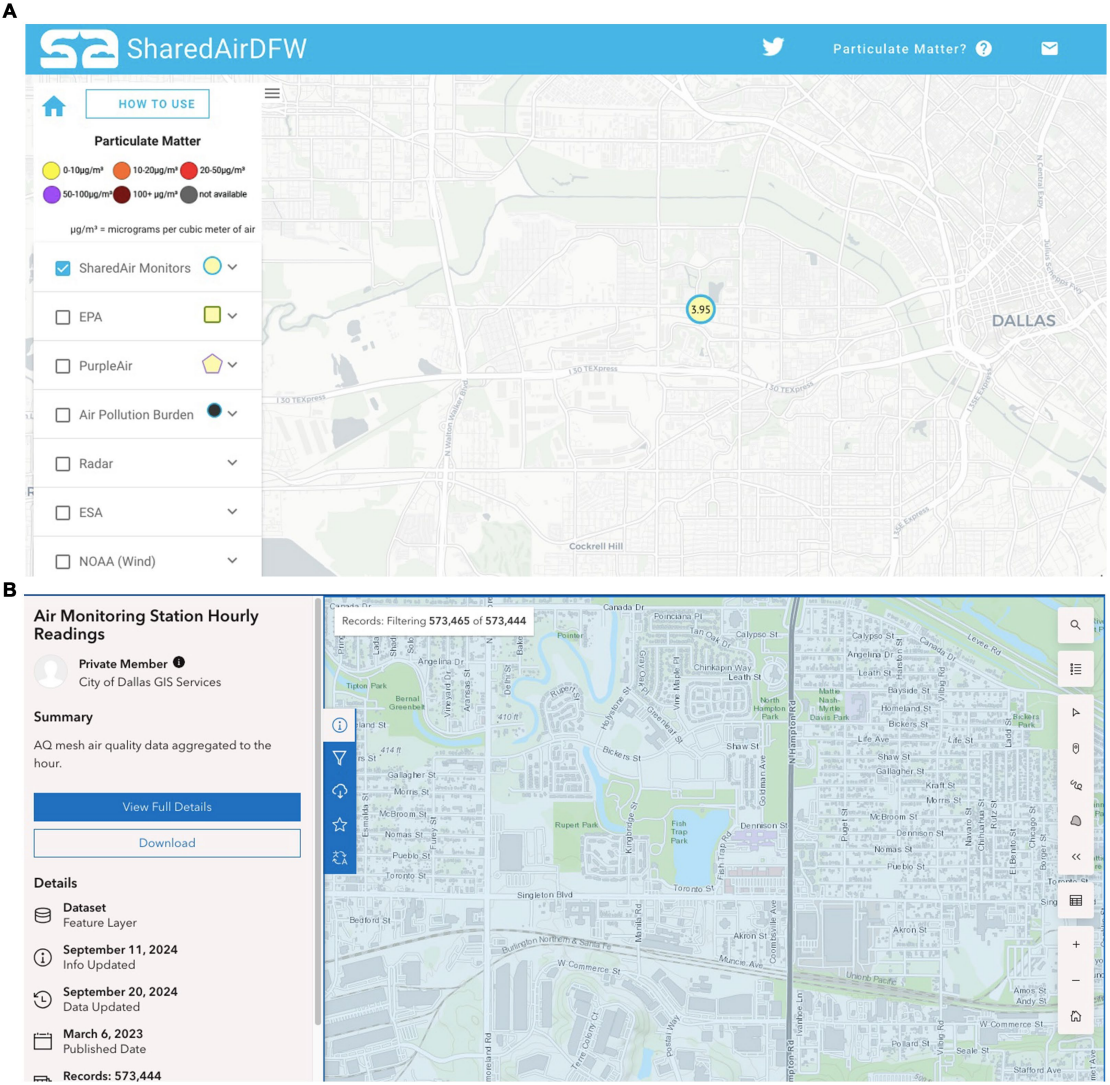
Low-cost sensors’ dashboards. Residents had access to the University of Texas at Dallas (UTD) SharedAirDFW dashboard **(A)** and raw hourly averages of PM_2.5_ (μg/m^3^) concentrations from the Toronto St. monitor. **(B)** Residents also had access to the City of Dallas community air monitoring dashboard and raw hourly averages of PM_2.5_ (μg/m^3^) concentrations from the Fish Trap and West Dallas multipurpose center monitors in West Dallas.

Residents were able to access hourly PM_2.5_ averages from the Toronto St. monitor via the SharedAirDFW dashboard (Figure 2A). The corrected hourly averages ranged from 2.82 to 961.78 μg/m^3^ during the study period. For comparison, the City of Dallas’ low-cost sensors in West Dallas—specifically, the Fish Trap monitor and the West Dallas Multipurpose Center (WDMPC) monitor—also reported raw and corrected hourly measurements (Figure 2B). Corrected hourly averages for the Fish Trap monitor ranged from 3.51 to 15.90 μg/m^3^, and from 3.54 to 18.07 μg/m^3^ for the WDMPC monitor. The nearest regulatory monitor, operated by the Texas Commission on Environmental Quality (TCEQ) and located approximately 4 miles away on Hinton Street, reported 1-h PM_2.5_ concentrations ranging from 0.69 to 37.99 μg/m^3^ and 24-h averages from 1.70 to 19.1 μg/m^3^. A visual comparison of the 1-h PM_2.5_ concentrations from all three low-cost sensors and the Hinton Street regulatory monitor is provided in Supplementary Figure 2.

## 4 Discussion

### 4.1 Application of CBPR

Low socioeconomic status Communities of Color have historically faced, and continue to face, disproportionate environmental exposures and disease burdens. This environmental health project, conducted in an EJ community located in West Dallas, is among the first of its kind, to our knowledge, in the Dallas-Fort Worth (DFW) industrial zoning area. Driven by residents’ environmental health concerns, our pilot project established a bidirectional partnership between the community and academia. Specifically, through collaboration with local community organizations, environmental advocacy groups, and academic researchers, our team examined local community residents’ risk perceptions and concerns about air quality, particularly focusing on air pollution burdens in the “Singleton Corridor” neighborhood and its health impact. The survey findings provide scientific evidence for developing a community-based action plan and establishing a foundation for future environmental health initiatives in West Dallas.

The systematic application of CBPR principles enabled successful project development and implementation while building community research capacity for future studies (e.g., prevention programs’ development). Several studies have highlighted the benefits of CBPR partnerships in EJ communities, primarily noting how CBPR partnerships have led to community-level action to improve the health and well-being of marginalized communities (6, 10–12, 51). Our academic-community partnership approach, adopted from our previous experience in a South Dallas EJ community (6), treated our community partners equally. We addressed community engagement through multiple mechanisms, which included providing CITI certification and IRB training for community volunteers, who then implemented culturally appropriate recruitment strategies such as door-to-door surveys. Key community stakeholders participated in all research phases, from developing bilingual survey instruments to selecting data collection methodologies (e.g., paper-based, and virtual platforms-based), and data interpretation in lay language for dissemination of findings across social media and community platforms. The achieved response rate of 40% in this predominantly minority community demonstrates the effectiveness of these community engagement strategies in building trust between community and researchers. This robust framework not only supported the current study but also established a foundation for future research initiatives tailored to community-identified needs and priorities.

The CBPR approach presented here serves as a template for how industrially burdened communities can gather the necessary information to combat regressive land use planning through comprehensive health impact data. There is continued opportunity across the DFW Metroplex to replicate this approach. Specifically, the Echo Heights and Northside communities in Fort Worth are laden with uncovered health concerns due to industrial pollution that need to be brought to light. Replicating this process in the second-largest city in the DFW can modify how the entire region treats the co-location of industrial facilities and neighborhoods, as the health outcomes analyzed by these studies become more widely understood.

### 4.2 Air pollution perception, beliefs, awareness, and concerns

The survey findings indicated heightened community awareness and concerns about air quality in the Singleton Corridor, with over 88% of residents identifying air pollution as a significant community problem and approximately 85% specifically attributing it to nearby industrial facilities. This high level of community air pollution awareness and concern aligns with documented EJ issues in similar communities (13). Additionally, studies have pointed out that the primary motivation for conducting community-based air monitoring was due to residents’ concerns for air pollution health risks and residing near potential pollution sources (14). Hence, our study examined available PM_2.5_ data from low-cost sensors in the “Singleton Corridor” community.

A number of studies have shown how even the perception of living in a polluted environment is harmful for health (15–18); and (19). In our study, residents demonstrated behavioral adaptations to perceived poor air quality, with 61.6% avoiding outdoor exercise and 68.6% refraining from opening windows. These modifications to daily activities suggest that air pollution remains a persistent environmental hazard in West Dallas, warranting timely development of community, and/or policy-based pollution interventions. The residents’ clear recognition of industrial sources as primary pollution stressor (84.9%) particularly emphasizes the need for targeted strategies in this EJ community.

Furthermore, survey results showed that a high proportion of West Dallas residents (84%) perceived air pollution as having adverse impacts on their and their family’s health. Specifically, residents identified air pollution as a critical health threat, which may cause or worsen respiratory-related chronic diseases (e.g., asthma) and allergic symptoms (difficulty breathing, headache, eye problems). Their environmental health risk perception may be shaped by various factors, including sociodemographic characteristics (e.g., age, literacy level), increasing public and social media attention, and direct individual experiences with air pollution exposure. Particularly, residents reported experiencing multiple pollution sources, with significant exposure to industrial emissions (79.1%), train-related pollution (66.3%), and traffic pollution (59.3%). This cumulative pollution exposure burden is concerning in the context of EJ communities, where environmental stressors often co-occur with pre-existing social and economic vulnerabilities in the community (20, 21).

### 4.3 Health impact concern

Additionally, survey results demonstrated a high prevalence of respiratory diseases and symptoms (Supplementary Table S4). The respiratory health burden in the Singleton Corridor substantially is higher than available state and national benchmarks. Current asthma prevalence (26.7%) was approximately 3–4 times higher than Texas and national rates. Similarly, lifetime asthma diagnosis rates (31.4%) were more than double the comparison benchmarks (22–24). In addition, 44.2% of respondents demonstrated medium-to-high risk for COPD. However, this proportion cannot be directly compared to diagnosed COPD prevalence rate due to methodological differences, it indicates a concerning finding that warrants further clinical evaluation and intervention for such residents. These respiratory health disparities became pronounced among certain demographic groups, including women, older adults, and Black/African American residents, consistent with broader EJ literature documenting disproportionate health impacts in minority and low-income communities (25–27, 46). The community’s health burden was further compounded by high rates of pre-existing conditions, with half of respondents reporting obesity, and elevated health risk behaviors such as smoking (22%). Moreover, the majority of residents (76.7%) reported moderate to severe stress levels, suggesting that high community risk perception may contribute to psychological burden, which aligns with previous research on environmental stressors in disadvantaged communities (28). This combination of environmental exposures, chronic health conditions, and psychological stress illustrates the cumulative health burden experienced by this community, where environmental stressors interact with and potentially exacerbate existing physical and psychological health challenges. These findings underscore the need for holistic approaches to environmental health interventions that address both the physiological and psychological dimensions of environmental exposure burden in this community.

Our analysis demonstrated a notable pattern in respiratory health outcomes across key demographic and socioeconomic subgroups, which is consistent with findings from prior environmental justice literature (29, 30, 45). Women reported higher prevalence of both lifetime asthma (77.8%) and current asthma (78.3%) relative to their representation in the sample, reflecting national trends of greater asthma burden among women (31). Obesity emerged as a significant risk factor, with 59.3% of lifetime asthma cases and 56.5% of current asthma cases reported among participants with a body mass index (BMI) ≥ 30, compared to an overall obesity rate of 54.7% in the sample. This association is particularly concerning in a community facing chronic socioeconomic and environmental stressors that may exacerbate both obesity and respiratory disease risk.

Smoking status demonstrated expected patterns. Specifically, the current and former smokers represent 40.7% of lifetime asthma, closely matching their representation in the overall sample (44.2%). However, the high prevalence of asthma among never-smokers (55.6% of asthma) indicates that environmental exposures in this community may be significant contributors to respiratory disease burden, independent of individual smoking behavior. Educational attainment level had inverse relationships with respiratory disease risk, with participants having less than high school education showing disproportionately higher rates of COPD risk (31.6% of medium-high risk cases vs. 20.9% of the sample). Employment status showed that those unable to work due to disability had 25.9% of lifetime asthma cases and 39.5% of medium-high COPD risk cases, indicating the bidirectional relationship between respiratory disease and economic hardship.

The sociodemographic patterns highlight that respiratory health disparities in the Singleton Corridor reflect the intersection of environmental exposures with existing social vulnerabilities, suggesting that effective interventions should be developed to address both environmental hazards and underlying socioeconomic determinants of health.

### 4.4 Low-cost sensors monitoring local PM_2.5_ levels

Low-cost sensors enable the measurement of neighborhood-level PM_2.5_ variation, at the hyperlocal level, that are available to residents in real-time (32–35). Additionally, regulatory sensors are prohibitively expensive for most non-governmental organizations, making low-costs sensors the most accessible option for delivering critical air quality information to at-risk communities (36, 37). PM_2.5_ (μg/m^3^) concentrations from the SharedAirDFW Toronto St. monitor indicated a broad range with several peak concentrations in the summer months which may indicate potential hotspots in the area. The City of Dallas’ two low-cost sensors reported similar ranges, lower than the Toronto St. monitor, with the highest concentrations in the summer months (July, August, and September). Notably, the Toronto St. monitor was located nearest the major roadway and industrial point sources. Due to variation in the sensor technologies, additional co-location experiments may be warranted. Moreover, residential access to daily levels reported through these different channels should be determined. The nearest regulatory monitor ~4 miles away (Hinton Street) did not show any exceedances of the current 24-h PM_2.5_ standard (35 μg/m^3^). However, the recent lowering of the annual PM_2.5_ standard, from 12.0 to 9.0 μg/m^3^, may impact EJ communities like West Dallas. Wang et al. (38) reported “uncaptured hotspots” with high percentages of minority populations, including Dallas, potentially misclassified as in attainment due to gaps in the current PM_2.5_ air monitoring network. Low-cost sensors may help to identify potential hotspots while providing neighborhood-level exposure data where regulatory monitors are sparse.

While low-cost sensors offer advantages in affordability and hyperlocal monitoring, they also present limitations that should be acknowledged. Sensor performance may vary depending on placement, environmental conditions (e.g., extreme heat or high relative humidity), and duration of deployment. PurpleAir sensors, in particular, are known to overestimate PM_2.5_ under humid conditions due to hygroscopic particle growth and sensor housing design. Although correction factors help mitigate these issues, they may not fully account for site-specific influences, especially in communities like West Dallas with complex pollution sources (e.g., asphalt plants, traffic). Sensor drift over time and variability between different sensor models can also affect long-term reliability. These limitations underscore the importance of correction algorithms, periodic calibration, and ideally, local co-location with regulatory monitors to validate findings.

### 4.5 Risk communication

Overall, survey results provide valuable insights into effective environmental risk communication strategies within this community. Respondents identified community organizations and clinical health professionals (e.g., doctors or nurses) as their most trusted sources of information about local environmental hazard risks and health consequences. This finding reflects the critical role local community organizations have played in recent years by leading environmental protection, advocating for policy changes, and fostering trust-based relationships with residents, which provide a solid foundation for implementing future community-based actions. The preference for clinical health professionals aligns with existing literature demonstrating public trust in healthcare providers for environmental health risk education (13, 39–41). Clinicians are uniquely positioned to serve as educators, alert practitioners, and advocates for discussing environmental health risks with individuals and communities (40). However, effective risk communication requires a feasible approach that bridges scientific environmental risk assessment (e.g., qualitative, and quantitative) with community understanding (13, 20, 42, 43). Citizen science emerges as a promising strategy to address this gap. By engaging community members directly in data collection, analysis, and interpretation, citizen science can provide a transformative approach to environmental health research by demystifying scientific risk assessment processes, enhancing community understanding of environmental health risks, and building research capacity within EJ communities.

### 4.6 Individual practice and future interventions

Risk perceptions are the critical driver for individual behavioral change. In West Dallas, many community residents reported adaptive strategies such as avoiding outdoor activities and keeping windows closed during periods of perceived poor air quality. These behaviors reflect the community’s concerns about air pollution in residents’ daily lives and reveal complex challenges in health-protective practices. Additionally, these individual adaptations may cause potential unintended consequences. Over 40% of survey respondents reported poor indoor air quality, suggesting that staying indoors does not effectively mitigate the adverse effects of air pollution on respiratory and other chronic diseases. On the contrary, such adaptation behaviors may inadvertently exacerbate social isolation and contribute to health disparities, particularly impacting physical activity and psychological well-being. Therefore, addressing environmental risk exposure in EJ communities exposes the significant limitations of individual-level interventions. Persistent industrial pollution may not be adequately mitigated through personal choices alone, as residents may face long-lasting structural barriers that constrain their capacity to reduce exposure risks (20). These systemic constraints, including limited socioeconomic resources and structural inequities, underscore the urgent need for comprehensive policy interventions.

A multi-level approach that integrates individual risk perception with broader environmental and social structures may provide a more nuanced strategy for addressing environmental health disparities. By simultaneously promoting individual behavioral adaptation and pursuing systemic changes (e.g., regulatory reforms, rezoning), the community stakeholders can develop more holistic and equitable environmental health strategies (44) providing a more comprehensive framework for understanding and addressing environmental health challenges.

This issue ultimately requires a multi-level intervention framework. Individual-level interventions may include improving residents’ environmental health knowledge, promoting behavior change (e.g., removing indoor pollution sources), conducting screenings for asthma or other respiratory conditions, and installing indoor air filtration systems. Community-level interventions could focus on environmental awareness campaigns, civic engagement, citizen science training, and the development of neighborhood-based air pollution monitoring networks. Policy-level interventions include rezoning, emissions regulation, and the removal or relocation of harmful industrial sources.

These interventions—particularly when paired with media advocacy—can increase public awareness of the environmental injustices experienced by fenceline communities and generate the political pressure needed to advance environmental equity at the city, state, and federal levels. Furthermore, the long-term impact of policy interventions, such as the removal of industrial sources, can be evaluated through follow-up studies that measure changes in local PM_2.5_ concentrations and community health outcomes. These comparisons offer a measurable framework to assess progress and can provide a data-driven foundation for reforming zoning policies and prioritizing environmental justice in urban planning.

### 4.7 Limitations

Several limitations should be described. The relatively small sample size (*n* = 86) limits the statistical power for conducting robust multivariate analyses, prevents calculation of precise confidence intervals, and may affect the representativeness of the findings within the broader Singleton Corridor community and their generalizability to other environmental justice communities. Although the 40% survey response rate demonstrates our effective community engagement strategies, the sample may not fully capture the demographic diversity or scope of health experiences across the entire neighborhood. In addition, the cross-sectional survey design may not be able to test causal relationships between perceived air pollution exposure and reported health outcomes. The self-reported health outcomes and air pollution perceptions introduces potential recall bias, social desirability bias and response bias, where those most concerned about environmental health issues may be more likely to participate and report symptoms. Furthermore, the increased community awareness of environmental issues may have influenced participants’ perception and reporting of health symptoms. Despite these limitations, this CBPR-based case study provides valuable baseline data and provides a foundation for future longitudinal research and development of interventions with larger, more representative samples that can better characterize the relationship between environmental exposures and health outcomes in this environmental justice community.

Additionally, while exact residential addresses were collected, the majority of participants lived within the same small geographic area, limiting spatial variability. As a result, we were unable to geocode health outcomes meaningfully or link them to individual-level PM_2.5_ exposure differences. This limitation reflects both the constrained spatial resolution of available exposure data and the geographic clustering of environmental injustice in this community. Future research should incorporate high-resolution exposure assessment and broader spatial sampling to enable finer-scale exposure-health analyses. Finally, while this study did not include multivariable models, we present descriptive analyses of health outcomes stratified by age group and length of residence in Supplementary Table S3 to offer initial insight into potential cumulative exposure patterns.

## 5 Conclusion

This study demonstrates the effectiveness of a CBPR framework in building a robust academic-community partnership within a historical EJ community of West Dallas, Texas. By engaging key stakeholders, including residents, community organization representatives, and academic researchers, throughout the project’s lifecycle, we developed a collaborative approach. This community-engaged strategy not only generated scientific data on resident health status but, more importantly, empowered residents to voice their lived experiences, perceptions, thoughts and concerns about local air quality and its adverse health impacts. Furthermore, the high prevalence of self-reported respiratory diseases, allergic symptoms and chronic disease addresses accumulative stressors faced by the EJ communities, highlighting the urgent need for comprehensive environmental health interventions. By identifying community concerns about air pollution and reporting disease prevalence, these initial findings can inform local policymakers and public health agencies in prioritizing mitigation strategies and allocating resources to communities most at risk Findings also illustrate how historical racial zoning and industrial proximity continue to disproportionately burden marginalized minority communities. This phenomenon will continue while industrial adjacencies to residential communities persist. However, individual behavioral changes alone are insufficient to address systemic environmental injustices. Relying solely on personal adaptations may overlook the deeper structural inequities that contribute to environmental health disparities. Future research should prioritize longitudinal studies that track the long-term health outcomes associated with environmental risk exposure. Additionally, there is a critical need to develop strategies that build community assets and environmental health literacy, enabling residents to effectively advocate for themselves and challenge systemic environmental inequities.

## Data Availability

The raw data supporting the conclusions of this article will be made available by the authors, without undue reservation.
