# Supplementary material for: The West Dallas environmental health project: the importance of community health experiences related to air pollution
Source: Front Public Health. 2025 Jul 25;13:1613899. doi: 10.3389/fpubh.2025.1613899 (PMC12331629; doi:10.3389/fpubh.2025.1613899)
Supplement: Supplementary file 1 [file Data_Sheet_1.docx]

***Supplementary Material***


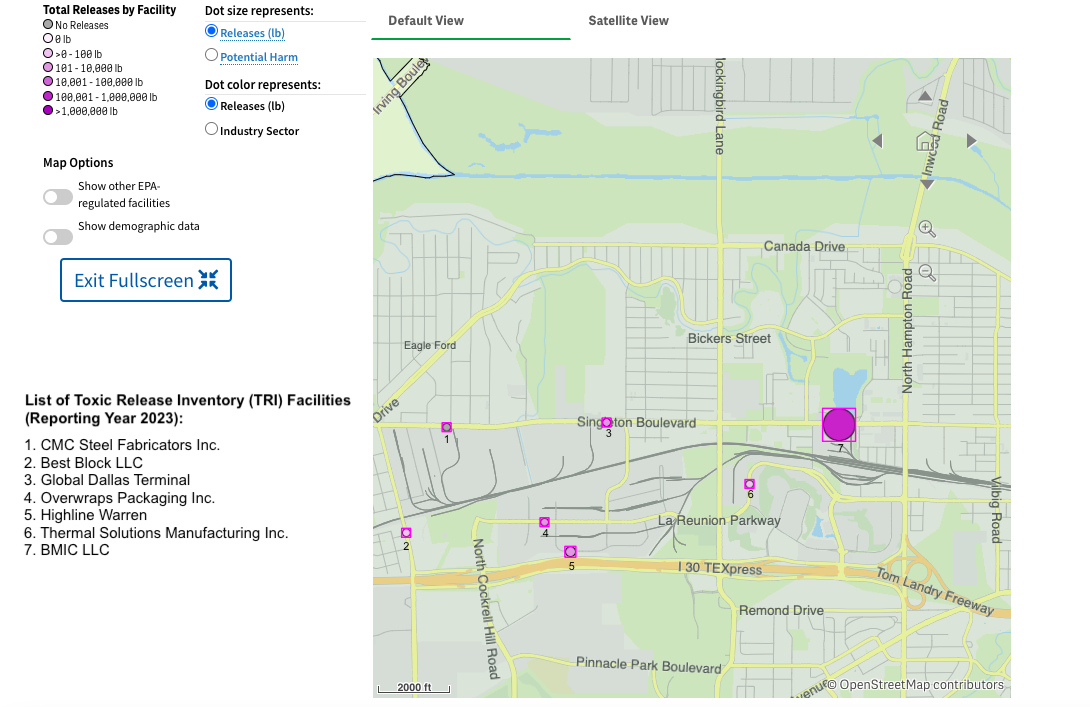


**Supplementary Figure 1.** **The U.S. Environmental Protection Agency’s Toxic Release Inventory (TRI) Toxics Tracker, Map of TRI Facilities (2023).** The purple circles represent toxic releases (in lb) by TRI facilities located in the Singleton Corridor, West Dallas, Texas.

**(A)**


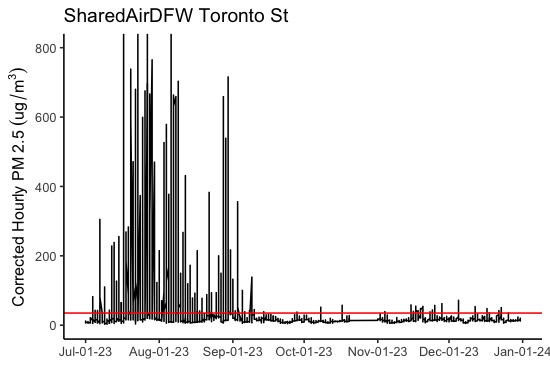


**(B)**


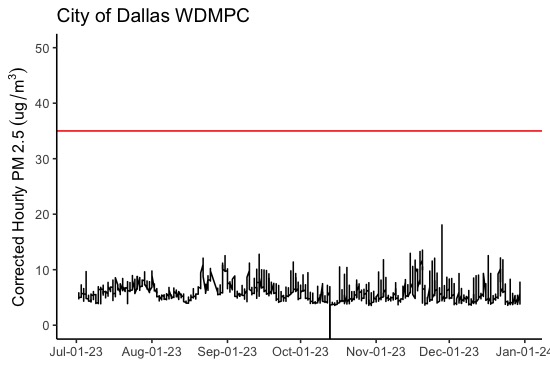


**(C)**


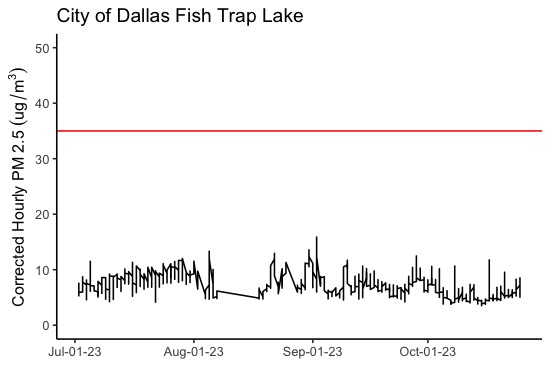


**(D)**


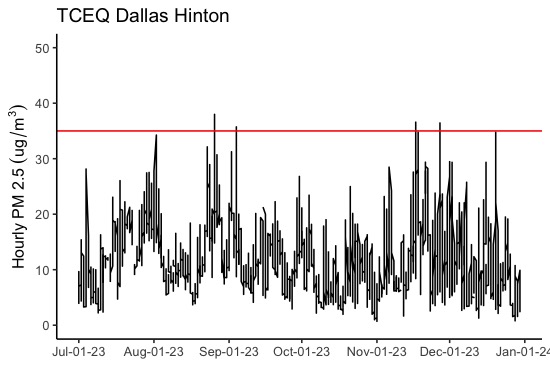


**Supplementary Figure 2.** **1-hour PM_2.5_ concentrations from low-cost sensors (*n* = 3) and regulatory monitor (*n* = 1) in West Dallas.** Hourly PM_2.5_ average concentrations plotted from SharedAirDFW’s Toronto St. sensor (A), City of Dallas’ WDMPC (B) and Fish Trap Lake (C) sensors, and the Texas Commission on Environmental Quality’s Dallas Hinton monitor (D). The red line indicates the US Environmental Protection Agency’s primary 24-hour PM_2.5_ standard of 35 μg/m^3^.

**Supplementary** **Table 1.** Socio-demographic profile of survey respondents (*n* = 86) in the Singleton Corridor, West Dallas, Texas, 2023.

| **Socio-demographic variables** | **Number (%)** |
| --- | --- |
| **Age** |  |
| 18-25 | 2 (2.35%) |
| 26-34 | 6 (7.96%) |
| 35-49 | 23 (27.06%) |
| 50-64 | 24 (28.24%) |
| 65 and above | 30 (35.29%) |
| **Gender** |  |
| Male | 23 (27.06%) |
| Female | 61 (71.76%) |
| Non-binary | 1 (1.18%) |
| **Education Attainment Level** |  |
| 0-11^th^ or no diploma | 16 (18.82%) |
| High school graduate or GED | 38 (44.71%) |
| Some college, but no degree | 20 (23.35%) |
| Associate degree | 3 (3.53%) |
| College degree | 5 (5.88%) |
| Advanced degree (e.g., MD, Ph.D., JD, Master’s Degree) | 2 (2.35%) |
| I prefer not to say | 1 (1.18%) |
| **Race/ethnicity** |  |
| Black/African American | 72 (84.71%) |
| Hispanic, Latino/a/x, Hispanic origin | 13 (15.29%) |
| Prefer not to say | N/A |
| **Marital Status** |  |
| Married | 12 (14.12%) |
| Single (not living with partner) | 47 (55.29%) |
| Single (living with partner) | 4 (4.71%) |
| Separated/Divorced | 9 (10.59%) |
| Widowed | 9 (10.59%) |
| Prefer not to say | 4 (4.71%) |
| **Employment Status** |  |
| Employed (including self-employed, part-time employment, full-time employment) | 30 (34.88%) |
| Unemployed | 9 (10.47%) |
| Homemaker or full-time family caregiver | 1 (1.16%) |
| Full time student | 2 (2.33%) |
| Unable to work due to disability or health condition (go to question #21) | 21 (24.42%) |
| Retired | 23 (26.74%) |
| **Insurance Status** |  |
| Bought coverage directly (e.g., Healthcare.gov, eHealthinsurance.com, directly from an insurance company like United Healthcare or BCBS or used an insurance agent/ broker) | 2 (2.35%) |
| Insurance through current or former employer union | 8 (9.41%) |
| Insured by my employer or union (including COBRA) | 4 (4.71%) |
| Insured through my spouse/partner’s employer or union | 1 (1.18%) |
| Medicaid, Medical assistance (MA), the children's health | 16 (18.82%) |
| Medicare, for people 65 and older | 29 (34.12%) |
| Any other types of health insurance coverage or health coverage plan | 5 (5.88%) |
| No health insurance | 20 (23.53%) |
| **BMI (based on CDC cut-off standard)** |  |
| <18.50 (underweight) | 1 (1.20%) |
| 18.5-24.9 (healthy weight) | 22 (26.51%) |
| 25.0 - <29.9 (overweight) | 16 (19.28%) |
| 30.0 and above (obesity) | 44 (53.01%) |
| **Smoking Status** |  |
| Never smoke | 48 (55.81%) |
| Current smoker | 19 (22.09%) |
| Former smoker | 18 (20.93%) |
| Prefer not to say | 1 (1.16%) |
| **Length of Residence** |  |
| ≤ 1 year | 4 (4.71%) |
| 2 - 5 years | 19 (22.35%) |
| 6 -10 years | 13 (15.29%) |
| 11 - 15 years | 14 (16.47%) |
| ≥ 16 years | 35 (41.18%) |

**Supplementary** **Table 2.** Air Pollution Exposure, Perceptions, and Attitudes of survey respondents (*n* = 86) in the Singleton Corridor, West Dallas, Texas, 2023.

| **Perceived Air Quality: Neighborhood and Home Rating** | |
| --- | --- |
| Rating on Air Quality in the Neighborhood, *(Low/Very low)* | 52 (60.47%) |
| Rating on Air Quality inside Home, *(Poor/Fair)* | 35 (40.70%) |
| **Perceptions and Awareness of Air Pollution Issues in the Community** | |
| Air pollution in my neighborhood is a problem,  *(Strongly Agree/Agree)* | 76 (88.37%) |
| Air pollution in my neighborhood is impacted by nearby industry,  *(Strongly Agree/Agree)* | 73 (84.88%) |
| Air pollution in my neighborhood affects my health or my family's health,  *(Strongly Agree/Agree)* | 72 (83.72%) |
| I avoid exercising outdoors because of air pollution, *(Often/Always)* | 53 (61.63%) |
| I avoid opening my windows because of air pollution, *(Often/Always)* | 59 (68.60%) |
| **Perceived Air Pollution Exposure Level** | |
| Exposure to Traffic, *(High/Very High)* | 51 (59.30%) |
| Exposure to Factories/Smokestacks, *(High/Very High)* | 68 (79.07%) |
| Exposure to Train, *(High/Very High)* | 57 (66.28%) |
| Exposure to other sources, *(High/Very High)* | 3 (3.49%) |
| **Concerns about Health Impact of Air Pollution** | |
| Worried that the air pollution from factories will cause Health Problem, *(Moderate/Extremely Concerned)* | 54 (62.79%) |
| **Beliefs on Health Impact of Air Pollution Sources and Conditions** | |
| **Believe that the following source of air pollution cause my health problems** |  |
| Traffic | 53 (61.63%) |
| Train | 59 (68.60%) |
| Factories/smokestacks | 75 (87.21%) |
| Other sources | 5 (5.81%) |
| **Think that air pollution may be caused or made worse for the following health conditions** (Top 6 health concerns) |  |
| Difficulty breathing | 62 (72.09%) |
| Cough/Cold | 56 (65.12%) |
| Asthma and other respiratory diseases | 54 (62.79%) |
| Headache | 49 (56.98%) |
| Eye problem | 45 (52.33%) |
| Allergic Reaction | 43 (50.00%) |

**Supplementary** **Table 3.** Health status by socio-demographic characteristics of survey respondents (*n* = 86) in the Singleton Corridor, West Dallas, Texas, 2023.

| **Socio-demographic Characteristic Variables** | **Lifetime asthma *** | **Current Asthma**** | **Respiratory diseases attack in past year** | **COPD^#^**  Medium or High Risk | **Perceived stress scale (PSS)**  Moderate or High Risk | **Nasal Allergy** | **Wheezing** | **Cough without weather impact** | **Cough with a phlegm (without cold)** | **Cough with a phlegm (first in the morning)** |
| --- | --- | --- | --- | --- | --- | --- | --- | --- | --- | --- |
| **Total**  Number (%) | 27 (31.40%) | 23 (26.74%) | 36 (41.86%) | 38 (44.19%) | 66 (76.74%) | 46 (53.49%) | 30 (34.88%) | 43 (50.00%) | 42 (48.84%) | 27 (31.40%) |
| **Age *(years)*** | | | | | | | | | | |
| 18-25 | 1 (50.0%) | 0 (0.00%) | 0 (0.00%) | NA^&^ | 2 (100%) | 0 (0.00%) | 0 (0.00%) | 2 (100%) | 1 (50.00%) | 1 (50.00%) |
| 26-34 | 3 (50.00%) | 1 (16.67%) | 0 (0.00%) | NA | 3 (50.00%) | 2 (33.33%) | 0 (0.00%) | 5 (83.33%) | 1 (16.67%) | 1 (16.67%) |
| 35-49 | 5 (21.74%) | 5 (21.74%) | 10 (43.48%) | NA | 18 (78.26%) | 13 (56.52%) | 8 (34.78%) | 12 (52.17%) | 10 (43.48%) | 5 (21.74%) |
| 50-64 | 8 (33.33%) | 8 (33.33%) | 11 (45.83%) | NA | 17 (70.83%) | 15 (62.50%) | 10 (41.67%) | 7 (29.17%) | 15 (62.50%) | 8 (33.33%) |
| 65 and above | 9 (30.00%) | 8 (26.67%) | 14 (46.67%) | NA | 25 (83.33%) | 15 (50.00%) | 12 (40.00%) | 16 (53.33%) | 15 (50.00%) | 12 (40.00%) |
| **Gender** | | | | | | | | | | |
| Male | 4 (17.39%) | 3 (13.04%) | 6 (26.09%) | 12 (52.17%) | 13 (56.52%) | 10 (43.48%) | 6 (26.09%) | 14 (60.87%) | 9 (39.13%) | 5 (21.74%) |
| Female | 21 (34.43%) | 18 (29.51%) | 28 (46.67%) | 25 (40.98%) | 51 (83.61%) | 34 (55.74%) | 23 (37.70%) | 28 (45.90%) | 32 (52.46%) | 21 (34.43%) |
| Non-binary | 1 (100%) | 1 (100%) | 1 (100%) | 1 (100%) | 1 (100%) | 1 (100%) | 1 (100%) | N/A | 1 (100%) | 1 (100%) |
| **Race/Ethnicity** | | | | | | | | | | |
| Black/African American | 24 (33.33%) | 21 (29.17%) | 31 (43.01%) | 35 (48.61%) | 57 (79.17%) | 42 (58.33%) | 27 (37.5%) | 35 (48.61%) | 37 (51.39%) | 24 (33.33%) |
| Hispanic (Latino/a/x) | 2 (15.38%) | 1 (7.69%) | 4 (30.78%) | 3 (23.08%) | 8 (61.54%) | 3 (23.08%) | 3 (23.08%) | 7 (53.85%) | 5 (38.46%) | 3 (23.08%) |
| **Education Attainment Level** | | | | | | | | | | |
| 0-11th grade/ no diploma | 3 (18.75%) | 3 (18.75%) | 7 (43.75%) | 12 (75.00%) | 9 (56.25%) | 6 (37.50%) | 6 (37.50%) | 8 (50.00%) | 6 (37.50%) | 4 (25.00%) |
| High school graduate/GED | 12 (31.58%) | 11 (28.95%) | 15 (39.47%) | 17 (44.74%) | 32 (84.21%) | 21 (55.26%) | 13 (34.21%) | 17 (44.74%) | 18 (47.37%) | 14 (36.84%) |
| Some college or associate degree | 10 (43.48%) | 7 (30.43%) | 9 (39.13%) | 5 (21.74%) | 18 (78.267%) | 12 (52.17%) | 7 (30.43%) | 13 (56.52%) | 12 (52.17%) | 7 (30.43%) |
| College degree and above | 1 (14.29%) | 1 (14.29%) | 4 (57.14%) | 3 (42.86%) | 5 (71.43%) | 5 (71.43%) | 4 (57.14%) | 4 (57.14%) | 5 (71.43%) | 2 (28.57%) |
| Prefer not to say | 0 (0.00%) | 0 (0.00%) | 0 (0.00%) | 1 (100%) | 1 (100%) | 1 (100%) | 0 (0.00%) | N/A | 1 (100%) | 0 (0.00%) |
| **Marital Status** | | | | | | | | | | |
| Married | 2 (16.67%) | 1 (8.33%) | 2 (16.67%) | 5 (41.67%) | 8 (66.67%) | 2 (16.67%) | 3 (25.00%) | 7 (58.33%) | 4 (33.33%) | 2 (16.67%) |
| Single | 19 (37.25%) | 16 (31.37%) | 24 (47.06%) | 21 (41.18%) | 40 (78.43%) | 31 (60.78%) | 20 (39.22%) | 23 (45.10%) | 26 (50.10%) | 19 (37.25%) |
| Separated/ Divorced/Widowed | 3 (16.67%) | 3 (16.67%) | 8 (44.44%) | 9 (50.00%) | 13 (72.22%) | 10 (55.56%) | 6 (33.33%) | 11 (61.11%) | 10 (55.56%) | 6 (33.33%) |
| Prefer not to say | 2 (50.00%) | 2 (50.00%) | 1 (25.00%) | 3 (75.00%) | 4 (100%) | 2 (50.00%) | 1 (25.00%) | 1 (25.00%) | 2 (25.00%) | 0 (0.00%) |
| **BMI** | | | | | | | | | | |
| <18.50 (Underweight) | 1 (100.00%) | 1 (100.00%) | 1 (100.00%) | 1 (100.00%) | 1 (100.00%) | 1 (100.00%) | 1 (100.00%) | N/A | 1 (100.00%) | 1 (100.00%) |
| 18.5 - 24.9 (Healthy weight) | 4 (18.18%) | 3 (13.64%) | 4 (18.18%) | 18 (81.82%) | 17 (77.27%) | 11 (50.00%) | 3 (13.64%) | 11 (50.00%) | 11 (50.00%) | 6 (27.27%) |
| 25.0 - <29.9 (Overweight) | 4 (25.00%) | 4 (25.00%) | 5 (31.25%) | 5 (31.25%) | 10 (62.50%) | 10 (62.50%) | 6 (37.50%) | 11 (68.75%) | 5 (31.25%) | 0 (0.00%) |
| 30.0 and above (Obesity) | 16 (36.36%) | 13 (29.55%) | 23 (52.27%) | 12 (27.27%) | 36 (81.82%) | 22 (50.00%) | 18 (40.91%) | 19 (43.18%) | 24 (54.55%) | 19 (43.18%) |
| **Smoking Status** | | | | | | | | | | |
| Current smoker | 6 (31.58%) | 4 (21.05%) | 9 (47.37%) | 11 (57.89%) | 15 (78.95%) | 12 (63.16%) | 6 (31.58%) | 9 (47.37%) | 10 (52.63%) | 7 (36.84%) |
| Former smoker | 5 (27.78%) | 4 (22.22%) | 8 (44.44%) | 10 (55.56%) | 14 (77.78%) | 11 (61.11%) | 9 (50.00%) | 7 (38.89%) | 14 (77.78%) | 10 (55.56%) |
| Never smoke | 15 (31.25%) | 14 (29.17%) | 18 (37.50%) | 17 (35.42%) | 35 (72.92%) | 23 (47.92%) | 15 (31.25%) | 26 (54.17%) | 17 (35.42%) | 10 (20.83%) |
| Prefer not to say | 1 (100.00%) | 1 (100.00%) | 1 (100.00%) | 0 (0.00%) | 1 (100.00%) | 0 (0.00%) | 0 (0.00%) | 1 (100.00%) | 1 (100.00%) | 0 (0.00%) |
| **Employment Status** | | | | | | | | | | |
| Employed | 7 (23.33%) | 6 (20.00%) | 12 (40.00%) | 5 (16.67%) | 24 (80.00%) | 19 (63.33%) | 11 (36.67%) | 17 (56.67%) | 12 (40.00%) | 7 (23.33%) |
| Unemployed | 2 (22.22%) | 2 (22.22%) | 3 (33.33%) | 2 (22.22%) | 7 (77.78%) | 3 (33.33%) | 3 (33.33%) | 2 (22.22%) | 5 (55.56%) | 4 (44.44%) |
| Homemaker or full-time family caregiver | 0 (0.00%) | 0 (0.00%) | 1 (100%) | 1 (100%) | 0 (0.00%) | 0 (0.00%) | 0 (0.00%) | N/A | 1 (100%) | 1 (100%) |
| Full-time Student | 2 (100%) | 0 (0.00%) | 0 (0.00%) | 0 (0.00%) | 1 (50.00%) | 0 (0.00%) | 0 (0.00%) | 1 (50.00%) | 1 (50.00%) | 1 (50.00%) |
| Unable to work due to disability or health condition | 7 (33.33%) | 7 (33.33%) | 9 (42.86%) | 15 (71.43%) | 16 (76.19%) | 10 (47.62%) | 7 (33.33%) | 8 (38.10%) | 13 (61.90%) | 6 (28.57%) |
| Retired | 9 (39.13%) | 8 (34.78%) | 11 (47.83%) | 15 (65.22%) | 18 (78.26%) | 14 (60.87%) | 9 (39.13%) | 15 (65.22%) | 10 (43.48%) | 8 (34.78%) |
| **Length of residency** | | | | | | | | | | |
| ≤1 year | 2 (50.00%) | 1 (25.00%) | 1 (25.00%) | 1 (25.00%) | 4 (100%) | 3 (75.00%) | 2 (50.00%) | 1 (25.00%) | 3 (75.00%) | 1 (25.00%) |
| 2-5 years | 9 (47.37%) | 7 (36.84%) | 7 (36.84%) | 6 (31.58%) | 13 (68.42%) | 9 (47.37%) | 5 (26.32%) | 9 (47.37%) | 9 (47.37%) | 5 (26.32%) |
| 6-10 years | 2 (15.38%) | 2 (15.38%) | 5 (38.46%) | 4 (30.77%) | 12 (92.31%) | 7 (53.85%) | 5 (38.46%) | 7 (53.85%) | 7 (53.85%) | 5 (38.46%) |
| 11-15 years | 5 (35.71%) | 5 (35.71%) | 7 (50.00%) | 8 (57.14%) | 12 (85.71%) | 9 (64.29%) | 5 (35.71%) | 4 (28.57%) | 6 (42.86%) | 5 (35.71%) |
| ≥16 years | 8 (22.86%) | 7 (20.00%) | 15 (42.86%) | 19 (54.29%) | 24 (36.36%) | 17 (48.57%) | 13 (37.14%) | 21 (60.00%) | 17 (48.57%) | 11 (31.43%) |

*Lifetime asthma is defined as having had asthma at some point in the life; **Current asthma is defined as having been diagnosed as asthma in their lifetime and also having asthma at the present time. ^#^ COPD denotes Chronic obstructive pulmonary disease; ^&^ The age groups for calculating COPD risk score are 40-49, 50-59, 60-69 and 70+;

**Supplementary** **Table 4.** Comparison of Respiratory Disease Prevalence: The Singleton Corridor Community vs. Dallas County, Texas State, and National Populations.

|  | Singleton Corridor | Dallas County | Texas state | National |
| --- | --- | --- | --- | --- |
| Current adult asthma rate | 26.7% | 14.0%* | 7.9%^ | 8.7%^#^ |
| Lifetime adult asthma rate | 31.4% | N.A. | 12.8%^ | 14.5%^#^ |
| COPD risk rate | 44.2% | N.A. | 5.1%^§^ | 6.4%^§^ |

* Data source: 2022 Dallas County Community Health Needs Assessment, Behavioral Risk Factor Surveillance System (BRFSS) 2016-2020.

^ Data source: Texas Behavioral Risk Factor Surveillance System (BRFSS) Public Use Data File, 2018-2022 and Statista, 2021.

# Data source: Centers for Disease Control and Prevention (CDC) surveillance data, and National Health Interview Survey 2022.

^§^ Data source: America’s Health Ranking, 2023 and CDC BRFSS, 2023. Specifically, Texas and National Chronic Obstructive Pulmonary Disease (COPD) prevalence was measured by prevalence of adults who reported ever being told by a health professional that they had COPD. The data has different methodologies from what we collected in the Singleton Corridor community health survey.

**Supplementary Material References**

America's Health Rankings. (2024). *Chronic obstructive pulmonary disease in Texas*. United Health Foundation. <https://www.americashealthrankings.org/explore/measures/COPD/TX> (Accessed June 10, 2025).

Centers for Disease Control and Prevention. (2024). *Most recent national asthma data*. <https://www.cdc.gov/asthma/most_recent_national_asthma_data.htm> (Accessed June 10, 2025).

Centers for Disease Control and Prevention. (2023). *2022: Percentage of persons with lifetime asthma by age*. <https://www.cdc.gov/asthma-data/nhis/2022-percentage-persons-lifetime-age.html> (Accessed June 10, 2025).

Dallas County Health and Human Services & Parkland Health. (2022). *2022 Dallas County Community Health Needs Assessment*. Dallas County. <https://www.dallascounty.org/Assets/uploads/docs/hhs/chna/CHNA-2022-WEB.pdf> (Accessed June 10, 2025).

Statista. (2021). *Lifetime asthma prevalence among U.S. adults in 2021, by state*. <https://www.statista.com/statistics/253902/us-states-with-highest-asthma-prevalence-among-adults/>

Texas Department of State Health Services. (2025). *Impact of asthma in Texas: 2025 report*. Texas Department of State Health Services, Center for Health Statistics, Austin, TX. <https://www.dshs.texas.gov/sites/default/files/CHI-Asthma/Docs/Reports/Impact-of%20Asthma-in-Texas-2025-Report.pdf> (Accessed June 10, 2025).
